# Impact of Janus Kinase Inhibition with Tofacitinib on Fundamental Processes of Bone Healing

**DOI:** 10.3390/ijms21030865

**Published:** 2020-01-29

**Authors:** Timo Gaber, Antonia Clara Katharina Brinkman, Justyna Pienczikowski, Karoline Diesing, Alexandra Damerau, Moritz Pfeiffenberger, Annemarie Lang, Sarah Ohrndorf, Gerd-Rüdiger Burmester, Frank Buttgereit, Paula Hoff

**Affiliations:** 1 Charité–Universitätsmedizin Berlin, Corporate Member of Freie Universität Berlin, Humboldt-Universität zu Berlin, and Berlin Institute of Health, Department of Rheumatology and Clinical Immunology, 10117 Berlin, Germany; 2 German Rheumatism Research Centre (DRFZ) Berlin, a Leibniz Institute, 10117 Berlin, Germany; 3 Endokrinologikum Berlin, 10117 Berlin, Germany

**Keywords:** tofacitinib, bone healing, hMSC-migration, chondrogenic differentiation, osteogenic differentiation, osteoclast differentiation

## Abstract

Both inflammatory diseases like rheumatoid arthritis (RA) and anti-inflammatory treatment of RA with glucocorticoids (GCs) or non-steroidal anti-inflammatory drugs (NSAIDs) negatively influence bone metabolism and fracture healing. Janus kinase (JAK) inhibition with tofacitinib has been demonstrated to act as a potent anti-inflammatory therapeutic agent in the treatment of RA, but its impact on the fundamental processes of bone regeneration is currently controversially discussed and at least in part elusive. Therefore, in this study, we aimed to examine the effects of tofacitinib on processes of bone healing focusing on recruitment of human mesenchymal stromal cells (hMSCs) into the inflammatory microenvironment of the fracture gap, chondrogenesis, osteogenesis and osteoclastogenesis. We performed our analyses under conditions of reduced oxygen availability in order to mimic the in vivo situation of the fracture gap most optimal. We demonstrate that tofacitinib dose-dependently promotes the recruitment of hMSCs under hypoxia but inhibits recruitment of hMSCs under normoxia. With regard to the chondrogenic differentiation of hMSCs, we demonstrate that tofacitinib does not inhibit survival at therapeutically relevant doses of 10–100 nM. Moreover, tofacitinib dose-dependently enhances osteogenic differentiation of hMSCs and reduces osteoclast differentiation and activity. We conclude from our data that tofacitinib may influence bone healing by promotion of hMSC recruitment into the hypoxic microenvironment of the fracture gap but does not interfere with the cartilaginous phase of the soft callus phase of fracture healing process. We assume that tofacitinib may promote bone formation and reduce bone resorption, which could in part explain the positive impact of tofacitinib on bone erosions in RA. Thus, we hypothesize that it will be unnecessary to stop this medication in case of fracture and suggest that positive effects on osteoporosis are likely.

## 1. Introduction

Today about 20% of the people in Germany are of age 67 or higher with an increasing frequency for the next decades [1]. In 2060 every third will be aged 67 or older [1]. This demographic development will lead to a higher incidence of surgical interventions (such as fracture treatment and arthroplasty) because of an impaired or delayed bone healing, degenerative joint diseases and osteoporosis. Moreover, patients suffering from inflammatory disorders like rheumatoid arthritis (RA) often exhibit delayed fracture healing [2,3,4,5,6,7,8,9,10], or even develop pseudarthrosis [8,9,10]. Additionally, glucocorticoids (GCs) and nonsteroidal anti-inflammatory drugs (NSAIDs), both being in daily clinical use for the treatment of RA, have been reported to hamper bone metabolism and fracture healing [11,12,13,14,15]. Another potent therapeutic agent in the treatment of RA is the anti-inflammatory drug tofacitinib [16]. Tofacitinib belongs to the drug family known as JAK inhibitors or Jakinibs which target the receptor-associated Janus kinases (JAK) and subsequently regulate their signaling via the signal transducer and activator of transcription (STAT) protein family [16,17]. Indeed, meanwhile tofacitinib belongs to the standard treatments used in RA and is part of national and international guidelines [18,19]. However, until now it is not clear how to manage the therapy with tofacitinib when fractures occur. In more detail, inhibition of the JAK/STAT-pathway using tofacitinib can be assumed to influence fundamental processes of bone regeneration such as inflammation-driven recruitment of human mesenchymal stromal cells (hMSCs), chondrogenesis, osteogenesis and osteoclastogenesis [20,21,22,23]. Tofacitinib is a well-known JAK inhibitor, which has been demonstrated potent anti-inflammatory effects in several animal models [24,25] and in clinical studies [26,27,28]. One of the beneficial effects of tofacitinib is the inhibition of radiographic structural damage [29,30]. The underlying mechanism could be the reduction of osteoclast formation leading to a reduced bone resorption. Both osteoclast formation and bone resorption are well-known features of the pathogenesis of RA and of bone remodeling during fracture healing [31,32]. 

However, tofacitinib has not been fully investigated for its regulatory effects on osteoblast and osteoclast activity especially under the microenvironmental conditions found during bone regeneration, where blood vessels are disrupted and supply of nutrients and oxygen is reduced [20,33]. Based on the beneficial effects of tofacitinib, we hypothesized that tofacitinib controls excessive initial inflammation and promotes bone regeneration by enhancing chondrocyte/osteoblast differentiation and activity and by suppressing osteoclast differentiation and activity. Therefore, in this study we aimed at examining the effects of tofacitinib on processes of bone regeneration focusing on the migration of hMSCs towards a tumor necrosis factor (TNF)α gradient. TNFα was chosen as one of the known highly secreted factors within the early inflammatory phase in fracture healing [34,35]. Furthermore, we examined the differentiation of hMSCs to chondrocytes and osteoblasts, and the differentiation of primary human monocytes towards osteoclasts under reduced oxygen availability mimicking the in vivo situation of the fracture gap [20,33].

## 2. Results

### 2.1. Tofacitinib Dose-Dependently Promotes the Recruitment of hMSCs under Hypoxia but Inhibits Recruitment of hMSCs under Normoxia

**Figure 1:**
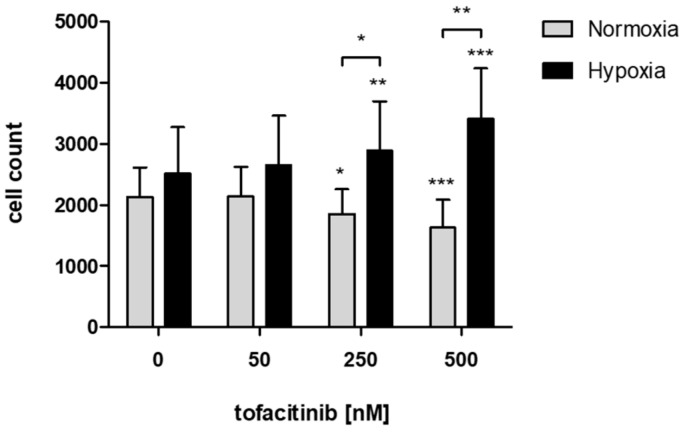
JAK inhibition by increasing doses of tofacitinib resulted in an increase of migrated hMSCs under hypoxic incubation conditions but reduced hMSC-migration under normoxia. Counts of migrated cells (*n* = 6; mean ± SEM; * *p* < 0.05, ** *p* < 0.01, *** *p* < 0.001; two-way ANOVA with Bonferroni post hoc test); asterisks above columns indicate comparison to the respective untreated control = 0 nM tofacitinib).

In order to study the effects of tofacitinib on the recruitment of hMSCs into the fracture hematoma, we demonstrate that JAK inhibition by increasing doses of tofacitinib resulted in an increase of migrated hMSCs towards an inflammatory milieu (5 ng/mL TNFα) under hypoxic incubation conditions but inhibited hMSC-migration under normoxia (Figure 1).

### 2.2. Tofacitinib Does Not Inhibit Survival and Chondrogenic Differentiation of hMSCs at Therapeutically Relevant Doses of 10–100 nM

**Figure 2:**
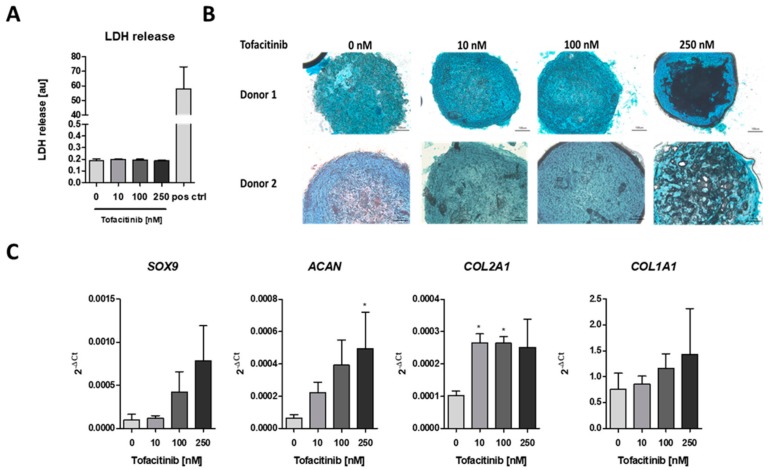
Tofacitinib did not inhibit survival and chondrogenic differentiation at therapeutic relevant doses of 10–100 nM. (**A**) LDH release was determined after 3 weeks of chondrogenic differentiation (*n* = 3; one-way ANOVA with Bonferroni post hoc test). (**B**) Alcian blue stainings of slices from cryo-preserved micro-mass cultures of chondrogenic differentiated hMSCs (2 of 4 donors, scale bars = 100 µm) (**C**) Chondrogenic marker gene expression for SOX9, ACAN, COL2A1 as well as osteogenic marker COL1A1 after 1 week of differentiation (*n* = 3; * *p* < 0.05; 1way ANOVA with Dunn’s multiple comparison post hoc test; asterisks above columns indicate comparison to the respective untreated control = 0 nM tofacitinib).

To analyze the impact of tofacitinib on chondrogenic differentiation, we first analyzed if cell survival is influenced by tofacitinib using the lactate dehydrogenase (LDH) release assay (Figure 2A). We observed no changes in LDH release between the doses tested. Moreover, LDH release was almost absent in comparison to the positive control after cell lysis using 2% Triton X-100. 

Using Alcian blue staining, we confirmed the chondrogenic differentiation of the hMSCs after three weeks of micro-mass culture under hypoxic conditions (2% O_2_) and tofacitinib treatment. In detail, we observed a similar Alcian blue staining of glycosaminoglycans (GAGs) after treatment with tofacitinib at doses up to 100 nM whereas at 250 nM the GAG content in the center of the micro-mass culture seemed to be reduced (Figure 2B). Moreover, chondrogenic marker gene expression of *SOX9*, *ACAN*, *COL2A1* increased with tofacitinib at least at the supra physiological doses (Figure 2C). Interestingly, also the expression of osteogenic *COL1A1* increased with increasing doses of tofacitinib, which may explain the GAG negative structures in the center of the micro-mass culture slides after treatment with 250 nM tofacitinib.

### 2.3. Tofacitinib Dose-Dependently Enhanced Osteogenic Differentiation of hMSCs 

**Figure 3:**
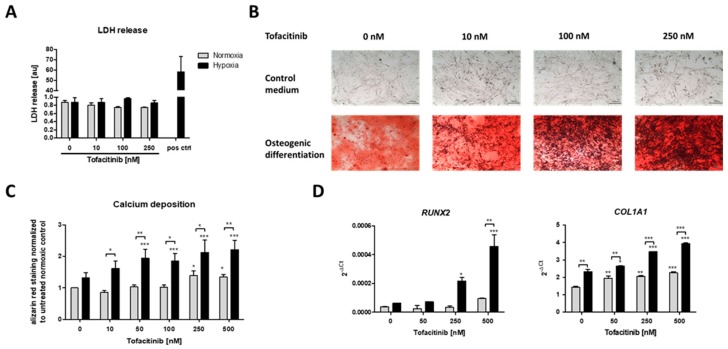
Calcium deposition and osteogenic marker gene expression as markers of osteogenic differentiation were enhanced by increasing doses of tofacitinib only under hypoxia. (**A**) LDH release after 3 weeks, (**B**) calcium deposits (scale bars = 100 µm) and (**C**) Alizarin Red staining after 3 weeks of osteogenic differentiation (*n* = 6; * *p* < 0.05, ** *p* < 0.01, *** *p* < 0.001; two-way ANOVA with Bonferroni post hoc test; asterisks above columns indicate comparison to the respective untreated control = 0 nM tofacitinib). (**D**) Osteogenic marker gene expression for RUNX2 and COL1A1 after 1 week of osteogenic differentiation (*n* = 3; * *p* < 0.05, ** *p* < 0.01, *** *p* < 0.001; two-way ANOVA with Bonferroni post hoc test; asterisks above columns indicate comparison to the respective untreated control = 0 nM tofacitinib).

After three weeks of osteogenic differentiation under normoxic (21% O_2_) or hypoxic conditions (1% O_2_) and tofacitinib treatment twice a day, we first analyzed if cell survival is influenced by tofacitinib during osteogenesis (Figure 3A). We observed no changes in LDH release with regard to (i) the incubation under either normoxic or hypoxic conditions and (ii) the doses of tofacitinib tested. Moreover, LDH release was almost absent in comparison to the positive control after cell lysis using 2% Triton X-100.

Using Alizarin Red staining, we confirmed the osteogenic differentiation of the hMSCs after three weeks of incubation under either normoxic (21% O_2_) or hypoxic conditions (1% O_2_) and tofacitinib treatment (Figure 3B,C).

JAK inhibition by increasing doses of tofacitinib resulted in an increase of calcium deposition as a marker of osteogenic differentiation under normoxia and to a higher extent under hypoxia (Figure 3B). Moreover, osteogenic marker gene expression of *RUNX2* and *COL1A1* increased with increasing doses of tofacitinib (Figure 3D).

### 2.4. Tofacitinib Reduces Osteoclast Differentiation and Activity

**Figure 4:**
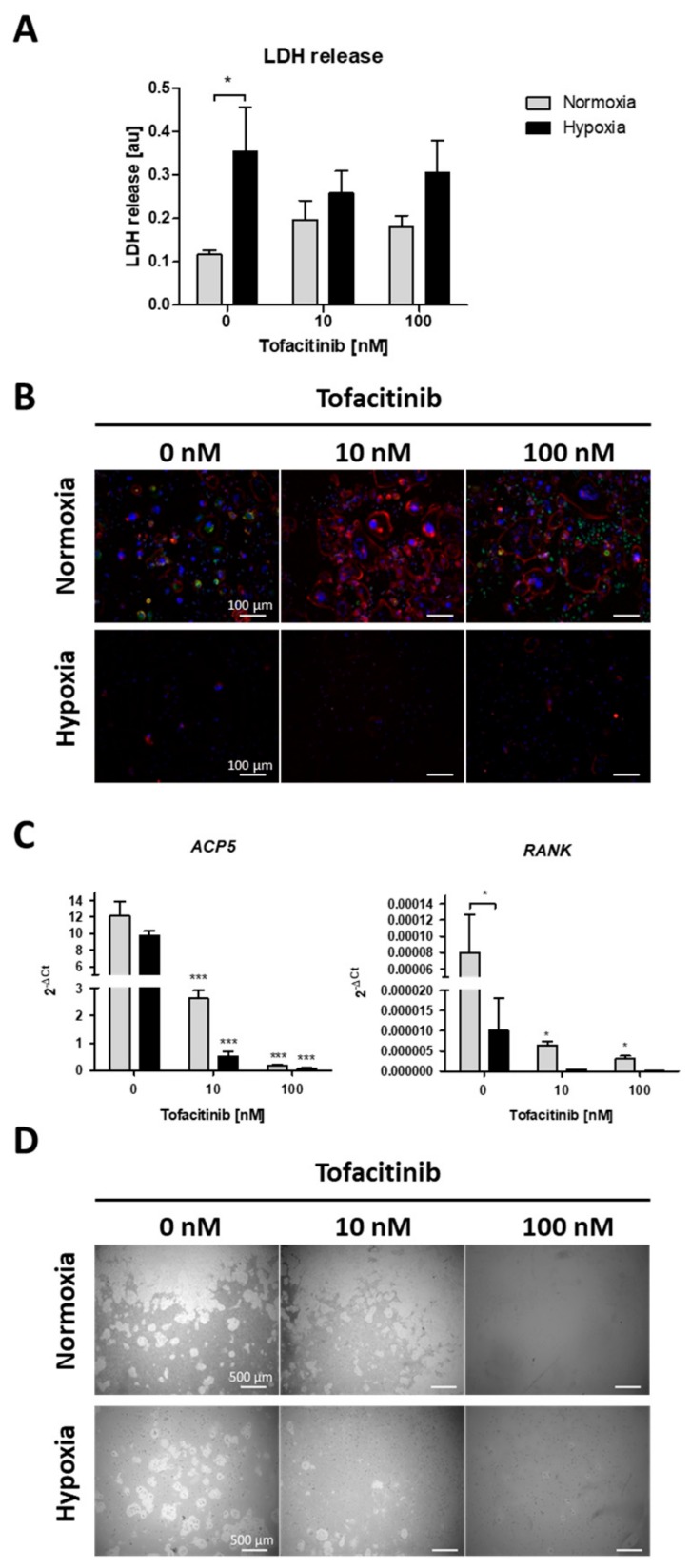
Tofacitinib reduces osteoclast differentiation and activity under normoxic and hypoxic conditions. (**A**) LDH release after 1 week (+3 days) of osteoclast differentiation and in the presence of 0, 10 and 100 nM tofacitinib under normoxic and hypoxic conditions (2% O_2_). (**B**) Representative experiment of *n* = 4. Fluorescence images of differentiated cells at day 21 in the presence of 0, 10 and 100 nM tofacitinib, stained with Phalloidin-TRITC for F-actin (red), DAPI (blue) and merged cathepsin K (green). Exemplary images representative for at least *n* = 4 in >3 independent experiments (scale bars = 100 µm). (**C**) Osteoclast marker gene expression for ACP5 and RANK after 1 week of osteoclast differentiation (*n* = 5; * *p* < 0.05, *** *p* < 0.001, two-way ANOVA with Bonferroni post hoc test; asterisks above columns indicate comparison to the respective untreated control = 0 nM tofacitinib). (**D**) Pit formation assay with osteoclasts differentiated on Corning^®^ Osteo Assay after Von Kossa staining. Exemplary images representative for at least *n* = 4 in >3 independent experiments (scale bars = 500 µm).

Focusing on the impact of tofacitinib on RANKL/M-CSF-induced osteoclast differentiation of monocyte under both normoxia and hypoxia, we first analyzed, if cell survival is influenced by oxygen availability and tofacitinib using the LDH release assay (Figure 4A). As a result, we observed a negative influence of hypoxia on cell vitality as demonstrated by a significant increase in LDH release of osteoclasts not treated with tofacitinib. However, we observed no changes in LDH release between the doses tested (Figure 4A). Analyzing osteoclast differentiation, we observed that RANKL/M-CSF-treated monocytes establish a confluent cell layer of many large and multinucleated osteoclasts (with horseshoe formation) and pre-osteoclasts (larger cells with one or two nuclei; (Figure 4B)). Activity status of osteoclasts could be determined by the expression of cathepsin K. With increasing doses osteoclasts become larger but are negative for the expression of cathepsin K, while bystander macrophages/pre-osteoclasts demonstrate strong expression of cathepsin K (Figure 4B).

Next, we analyzed the expression of osteoclast specific marker genes for tartrate-resistant acid phosphatase type 5 (*ACP5*) and receptor activator of NF-κB (*RANK*) (Figure 4C). Again, we observed a decrease in the expression of osteoclast specific marker genes with increasing doses of tofacitinib (at 10 nM and 100 nM) and by cells incubated under hypoxic conditions, at least in terms of *RANK* expression.

Finally, we analyzed the resorption activity of osteoclast by resorption pit assay (Figure 4D). We observed a reduction in pit formation by cells incubated under hypoxic conditions and with increasing doses of tofacitinib (at 10 and 100 nM).

## 3. Discussion

Inflammatory disorders like RA are associated with different co-morbidities like osteoporosis or diabetes mellitus [36,37,38,39]. A concomitant therapy with glucocorticoids is often required, but does potentially contribute to the development of osteoporosis and diabetes mellitus. The inflammatory disease itself, but also the therapy with glucocorticoids and a potentially co-existing diabetes mellitus collectively lead to impaired bone quality [36,37,38,39,40,41]. Thus, fractures and delayed fracture healing occur more often than in healthy people [1,2,3,4,5,6,7,8,9]. Tofacitinib is a potent anti-inflammatory therapeutic agent, which is successfully used to treat patients with RA [16]. It helps to taper glucocorticoids in patients with RA, and it has been shown to inhibit the radiographic progression of the disease [29,30]. As the local mechanism of maturation of erosions systemically contributes to osteoporosis development [42,43], an anti-osteoporotic effect can by hypothesized. However, patients with inflammatory diseases often interrupt their anti-inflammatory therapy after fracture. 

In the fracture gap cells promoting bone healing such as hMSCs face restricted conditions such as hypoxia due to interrupted blood supply and enhanced metabolic activity [20,34,35,44,45]. Previously, we have demonstrated, that hypoxia promotes osteogenesis in hMSCs [46], which indicates a supportive role of the restrictive hypoxic microenvironment in the fracture gap to initiate bone healing. Now, we here also demonstrate that JAK inhibition by tofacitinib dose-dependently promotes the recruitment of hMSCs to the hypoxic conditions found in the fracture gap (Figure 1). In addition, tofacitinib does not suppress chondrogenic differentiation of hMSCs and their survival at therapeutically relevant doses of 10–100 nM (Figure 2) and under hypoxic conditions such as found in the fracture gap [20,44,45]. Furthermore, hypoxia in conjunction with the inhibition of JAK/STAT-signaling promotes osteogenic differentiation of hMSCs (Figure 3). Supporting our latter observations, Levy et al. previously demonstrated that inhibition of STAT3 signaling pathway accelerates and augments BMP2- and BMP4-induced osteogenic differentiation of hMSCs [47]. 

In summary, after the enhanced recruitment of hMSCs to fracture site, osteogenic differentiation is promoted by tofacitinib while not compromising chondrogenic differentiation leading to the suggestion, that tofacitinib does not negatively impact fracture healing.

From the clinical point of view and based on our findings, we hypothesize patients with inflammatory diseases not to have necessarily pause tofacitinib treatment during fracture healing or to restart the therapy after a pause liberally at least in situations, when the patients feel a flare coming. However, this hypothesis needs to be proven in a clinical study. Patients would benefit from tofacitinib treatment firstly by achieving a stable disease activity/remission, secondly by promoting the recruitment of hMSCs to the hypoxic fracture site—the progenitors of osteoblasts—and thirdly by an accelerated osteogenic differentiation needed for fracture healing. Our data indicate that, tofacitinib does not interfere with the bone healing process at the stage of chondrocyte differentiation cartilaginous (soft) callus formation and the endochondral ossification and osteogenic differentiation for bony (hard) callus formation. Right now, tofacitinib is only allowed to be used in adults. If in future a treatment should be possible in children [48], we would expect from our data that also the length growth is not affected by tofacitinib: as the length growth is based on the mechanism of endochondral ossification and chondrogenic differentiation which was not negatively affected by tofacitinib. In contrast, tofacitinib dose-dependently enhanced osteogenic differentiation of hMSCs. This clearly shown effect again points in direction of safety of tofacitinib treatment also after a fracture occurred. 

As we know that radiographic progression of RA is inhibited by tofacitinib [29,30] and osteoclasts are involved in the emergence of erosions, we hypothesized that osteoclasts are negatively influenced by tofacitinib. Indeed, we could demonstrate reduced osteoclast differentiation and activity under normoxic and hypoxic conditions mediated by tofacitinib (Figure 4). Murakami et al. reported an inhibition of osteoclastogensis via baricitinib, another JAK inhibitor via suppressing RANKL expression on osteoblasts [49]. These two effects may go hand in hand and could explain the anti-erosive effect and also systemic anti-osteoporotic impact [29,30].

We conclude from our data, that tofacitinib may influence bone healing by promoting hMSC-recruitment into the hypoxic microenvironment of the fracture gap, may not interfere with the cartilage formation during the soft callus phase of fracture healing. We assume that tofacitinib may promote bone formation and reduce bone resorption which could in part explain the positive impact on bone erosions by tofacitinib. Thus, we hypothesize that it will be unnecessary to stop this medication in case of fracture and suggest that positive effects on osteoporosis are likely.

## 4. Materials and Methods

### 4.1. Antibodies, Growth Factors and Inhibitors

Antibodies to determine hMSC surface marker profile against CD73, CD90, CD105, CD34, CD45, CD20, CD14 and HLA-DR were purchased from Miltenyi Biotec (Miltenyi Biotec, Bergisch Gladbach, Germany). Anti-Cathepsin K antibody was purchased from Abcam (Abcam plc, Cambridge, UK), Phalloidin–Tetramethylrhodamine B isothiocyanate (Phalloidin-TRITC) was purchased from Sigma-Aldrich Chemie GmbH (Sigma-Aldrich Chemie Gmbh, Munich, Germany) and secondary goat anti-rabbit IgG was purchased from Thermo Fisher Scientific (Thermo Fisher Scientific, Waltham, MA, USA). Recombinant human receptor activator of NF-κB ligand (RANKL) and recombinant human macrophage colony-stimulating factor (M-CSF) were purchased from PeproTech (PeproTech, Rocky Hill, NJ, USA) and Miltenyi Biotec (Miltenyi Biotec, Bergisch Gladbach, Germany), respectively. Tofacitinib was obtained from Sigma-Aldrich Chemie Gmbh (Sigma-Aldrich Chemie Gmbh, Munich, Germany).

### 4.2. Isolation and Incubation of Bone Marrow-Derived hMSCs

Primary hMSCs were isolated from bone marrow of patients undergoing total hip replacement. Bone marrow was provided by the Center for Musculoskeletal Surgery (Charité-Universitätsmedizin Berlin). Study design and experimental approach were approved by the local Ethics Committee (Charité-Universitätsmedizin Berlin) and were conducted according to the Helsinki Declaration (ethical approval EA1/012/13, January 2013).

After surgical collection of bone marrow, the material was transferred to the laboratory using 50 mL reaction tubes (Sarstedt, Nümbrecht, Germany) and subsequently seeded under sterile conditions into a 175 cm^2^ cell tissue flask (Greiner Bio-One International GmbH, Kremsmünster, Austria) containing DMEM+GlutaMAX^TM^ (Thermo Fisher Scientific, Waltham, MA, USA) supplemented with 10% fetal calf serum (FCS; Biowest, Nuaillé, France), 1% Penicillin/Streptomycin (Thermo Fisher Scientific, Waltham, MA, USA) and 20% StemMACS™ MSC Expansion Media Kit XF (Miltenyi Biotec, Bergisch Gladbach, Germany). After two hours of cultivation in a humidified atmosphere (37 °C, 5% CO_2_), we discarded the supernatant including medium and remaining bone marrow, while adherent bone marrow-derived cells were washed twice with phosphate-buffered saline (PBS) with a pH of 7.4. Cells were maintained in the respective medium until passaging. Medium exchange was performed weekly. For the experiments, cells in passage 3–6 were used. 

### 4.3. Characterization of hMSCs

**Figure 5:**
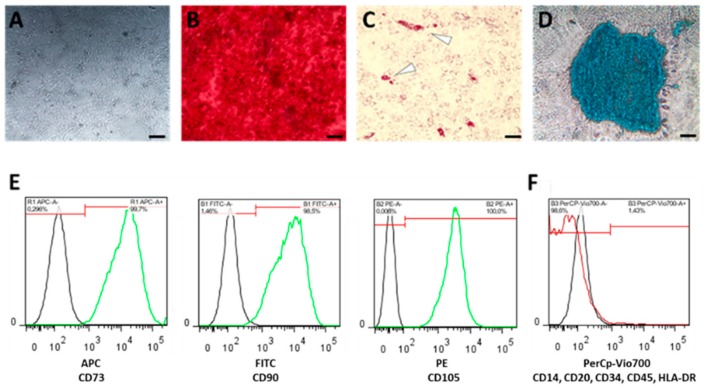
Bone marrow-derived human mesenchymal stromal cells (hMSCs) were characterized (**A**) by plastic adherence, and by their differentiation capacity towards (**B**) osteogenesis using Alizarin Red staining, (**C**) adipogenesis using Oil-Red-O staining, (**D**) chondrogenesis using Alcian Blue staining (scale bars = 200 µm), and (**E**) by the expression of surface marker CD105, CD90 and CD73, but the lack of (F) CD14, CD20, CD34, HLA-DR and CD45 expression.

For differentiation purposes, we seeded hMSCs at a density of 1 × 10^4^ cells/well in 96-well plates (Greiner Bio-One International GmbH, Kremsmünster, Austria) in a humidified atmosphere (37 °C, 5% CO_2_) (Figure 5A). After one day of incubation, we discarded the medium and added the appropriate differentiation medium, which was changed weekly.

For osteogenic differentiation, we incubated the seeded hMSCs in StemMACS^TM^ OsteoDiff (Miltenyi Biotec, Bergisch Gladbach, Germany) supplemented with 1% Penicillin/Streptomycin (Thermo Fisher Scientific, Waltham, MA, USA). After three weeks, adherent cells were fixed in 4% paraformaldehyde (PFA; Sigma-Aldrich Chemie Gmbh, Munich, Germany) for 15 min at room temperature, washed with PBS and stained with 0.5% Alizarin Red S (Sigma-Aldrich Chemie Gmbh, Munich, Germany) for another 15 min at room temperature. After the final washing step with ddH_2_O we analyzed and documented calcium deposition by microscopy (Figure 5B). 

For adipogenic differentiation, we incubated the seeded hMSCs for 3 weeks in StemMACSTM AdipoDiff (Miltenyi Biotec, Bergisch Gladbach, Germany). After incubation, hMSCs were fixed in 4% PFA for 15 min at room temperature, washed with 60% isopropanol and subsequently stained with 60% Red Oil O working solution solved in ddH_2_O (Sigma-Aldrich Chemie Gmbh, Munich, Germany; freshly prepared and filtered (0.45 µm); stock solution: 0.3% Red Oil O solved in 100% isopropanol) for 15 min at room temperature and washed again with 60% isopropanol. Finally, ddH2O was added and red stained lipid droplets were analyzed by microscopy (Figure 5C).

For chondrogenic differentiation, we incubated 2 × 10^4^ hMSCs per conical well for 3 weeks in StemMACS^TM^ ChondroDiff (Miltenyi Biotec, Bergisch Gladbach, Germany) in a conical 96-well plate under hypoxic conditions (2% O_2_). Cell pellets were centrifuged daily for 10 min at 400× *g* (Figure 5D). Resulting micro-mass cultures were washed with ddH_2_O and stained with 100 µL Alcian Blue solution (Sigma-Aldrich Chemie Gmbh, Munich, Germany) over night. Stained pellets were washed twice, air-dried and embedded in SCEM embedding medium (Section-Lab Co. Ltd., Hiroshima, Japan). We prepared slices of 7 µm using a microtome and analyzed and documented them by microscopy (Figure 5D).

Phenotypic characterization of hMSCs was conducted according to the manufacturers’ instruction using the MSC Phenotyping Kit (Miltenyi Biotec, Bergisch Gladbach, Germany) (Figure 5E,F) using the MACSQuant Analyzer (Miltenyi Biotec, Bergisch Gladbach, Germany).

Only cells which (i) differentiated towards adipogenic, chondrogenic and osteogenic lineage and (ii) fulfilled the relevant surface marker profile (CD73+, CD90+, CD105+; CD34-, CD45-, CD20-, CD14-, HLA-DR-) were used for the subsequent experiments.

### 4.4. Migration Assay of hMSCs

**Figure 6:**
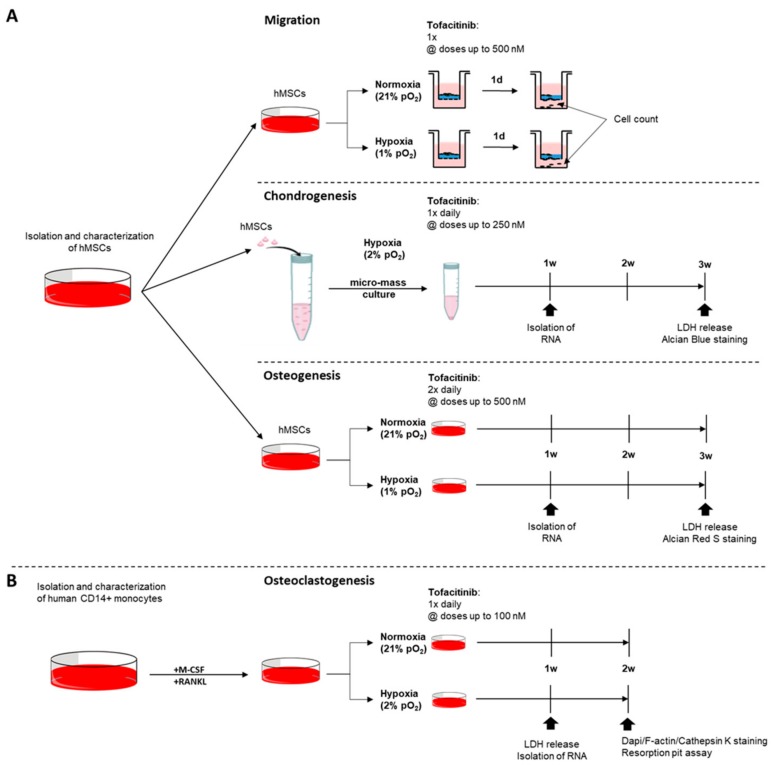
Experimental design. Assays on the impact of tofacitinib on (**A**) hMSCs with focus on (i) migration, (ii) chondrogenic differentiation, and (iii) osteogenic differentiation and (**B**) monocyte to osteoclast differentiation taking into account the availability of oxygen.

In order to study the effects of tofacitinib on hMSC-migration towards TNFα gradient, we seeded fully characterized hMSCs (5 × 10^4^ cells/well) in the presence of varying doses of tofacitinib (0, 50, 250, 500 nM) on a 0.1% gelatin-coated trans-well system for 24 h under normoxia and hypoxia, respectively (Figure 6A). Migrated cells were analyzed by cell staining using hemacolor staining (Sigma-Aldrich Chemie Gmbh, Munich, Germany) and counting using flow cytometry (MACSQuant Analyzer; Miltenyi Biotec, Bergisch Gladbach, Germany).

### 4.5. Analysis of Cell Survival Using Lactate Dehydrogenase (LDH) Release Assay

The LDH assay was conducted using the Cytotoxicity Detection Kit (Sigma-Aldrich Chemie Gmbh, Munich, Germany) with supernatants after week 3 for chondrogenesis and osteogenesis and after week 1 for osteoclastogenesis (Figure 6). OD-values were measured using a standard plate reader at a wavelength of 490 nm (reference wavelength 630 nm). Assay was performed in triplicates in two independent experiments. Positive control was obtained by cell lysis using 2% Triton X-100 (Sigma-Aldrich Chemie Gmbh, Munich, Germany). Tofacitinib treatment during chondrogenic differentiation

For analyzing the impact of tofacitinib on chondrogenic differentiation, hMSCs were incubated as described above and pellets were treated once daily (Figure 6A). Resulting micro-mass were analyzed by LDH release, Alcian Blue solution and the expression of chondrogenic marker genes including aggrecan (*ACAN*), Collagen type II alpha 1 chain (*COL2A1*) and SRY-box transcription factor 9 (*SOX9*).

### 4.6. Osteogenic Differentiation, Visualization and Quantification of Alizarin Red S Staining

For osteogenic differentiation, hMSCs (1 × 10^4^/well in a flat-bottom 96-well plate) were cultivated in StemMACS^TM^ OsteoDiff (Miltenyi Biotec, Bergisch Gladbach, Germany) supplemented with 1% Penicillin/Streptomycin (Gibco, Waltham, MA, USA) for three weeks with medium change performed twice daily with the respective varying concentrations of tofacitinib (Sigma-Aldrich Chemie Gmbh, Munich, Germany) (Figure 6A). Cells seeded were incubated under normoxic and hypoxic conditions. Afterwards cells were washed with PBS, fixated with 4% PFA for 15 min at room temperature, washed with PBS again and stained with 0.5% Alizarin Red S (Sigma-Aldrich Chemie Gmbh, Munich, Germany) solved in ddH_2_O for another 15 min at room temperature. Calcium deposition was analyzed microscopically. 

For quantification, Alizarin Red S staining was dissolved by the addition of/incubation with 10% cetylpyridinium chloride (Sigma-Aldrich Chemie Gmbh, Munich, Germany) dissolved in PBS on a shaker at 300 rpm for 15 min. Supernatants were transferred onto an optical 96-well EIA plate (Corning GmbH, Kaiserslautern, Germany) and quantified using a plate reader at a wavelength of 562 nm (reference wavelength 630 nm). Data were normalized to the OD values of the normoxic untreated control and the assay was performed in triplicates in two independent experiments.

### 4.7. Monocyte Isolation and Osteoclast Differentiation

Human peripheral blood was obtained from healthy donors with approval of the local Ethics Committee (Charité-Universitätsmedizin Berlin) and were performed according to the Helsinki Declaration (ethical approval EA1/207/17, approved October 2017 and EA1/367/14, approved January 2015). Peripheral blood mononuclear cells from buffy coats were isolated by density gradient centrifugation using Ficoll-Paque^TM^ Plus technique (GE Healthcare Europe GmbH, Freiburg, Germany). CD14+ monocytes were enriched up to 99% purity and >95% viability (as verified by flow cytometry) by MACS using anti-human CD14 conjugated magnetic beads (Miltenyi Biotec, Bergisch Gladbach, Germany). 

Monocytes were cultured at 1 × 10^7^ cells/ml in αMEM (Thermo Fisher Scientific, Waltham, MA, USA) supplemented with 1% Penicillin/Streptomycin (Thermo Fisher Scientific, Waltham, MA, USA), 100 ng/ml RANKL (PeproTech, Rocky Hill, NJ, USA) and 50 ng/mL M-CSF (Miltenyi Biotec, Bergisch Gladbach, Germany) and varying doses of tofacitinib (0, 10, 100 nM; Sigma-Aldrich Chemie Gmbh, Munich, Germany) (Figure 6B). 100 µL of cell suspension was incubated under normoxic and hypoxic conditions in 96-well flat bottom LUMOX^TM^ plates (Sarstedt, Nümbrecht, Germany). Fully supplemented αMEM was refreshed every three days for 3–4 weeks and differentiation was monitored by microscopy. 

### 4.8. Immunofluorescence Staining

For osteoclast detection, anti-rabbit cathepsin K antibody, conjugated Phalloidin-TRITC for F-actin staining and 4,6-Diamidino-2-phenylindoline (DAPI) for staining of nuclei were used after 3 weeks of differentiation and incubation with tofacitinib (doses as indicated in the figure legends). To this end, cells were washed with PBS and fixed with 4% PFA for 5–8 min at room temperature. Fixed cells were washed with PBS (3×) and permeabilization using 1 × PBS/0.1% Tween^®^ 20 (MP Biomedicals, LLC, Irvine, CA, USA) for 10 min. Non-specific binding was blocked with PBS/5% FCS for 30 min at room temperature. Subsequently, cells were washed 3× using 1× PBS/0.1% Tween^®^ 20 and incubated with the primary antibody, anti-cathepsin K (1:250, diluted in 1× PBS/5% FCS/0.1% Tween^®^ 20) for 30 min. After washing with 1× PBS/0.1% Tween^®^ 20, cells were incubated with the secondary Alexa 488-conjugated anti-rabbit antibody (1:500, diluted in 1× PBS/5% FCS/0.1% Tween^®^ 20) for 30 min. For visualization of F-actin, cells were stained using Phalloidin-TRITC (Sigma-Aldrich Chemie Gmbh, Munich, Germany; 50 µg/mL at 1:20 in 1× PBS/5% FCS/0.1% Tween^®^ 20) for 45 min at room temperature protected from light. Cells were washed using 1× PBS/0.1% Tween 2 and stained for nuclei using DAPI (Sigma-Aldrich Chemie Gmbh, Munich, Germany; 1 µg/mL in 1× PBS/5% FCS/0.1% Tween^®^ 20) for 10 min protected from light. Cells were washed using 1× PBS/0.1% Tween^®^ 20 and covered with PBS. Pictures were taken with a fluorescence microscope BZ 9000 (Keyence Deutschland GmbH, Neu-Isenburg, Germany) using lasers of specific wavelengths. The assay was performed in duplicates and at least two pictures per sample were analyzed.

### 4.9. Resorption Pit Assay

After isolation, monocytes were seeded in Corning^®^ Osteo Assay wells and differentiated for 21 days with RANKL and/or M-CSF changing the medium every 3 days and treated with varying doses of tofacitinib (0, 10, and 100 nM). Analysis of resorption pits and resorption efficiency was performed via Von Kossa. For visualization, Corning^®^ Osteo Assay wells (Corning GmbH, Kaiserslautern, Germany) with seeded cells were washed with PBS after 21 and fixed for 10 min in 4% PFA. After washing in ddH_2_O, cell layers were treated with 1% silver nitrate solution (Sigma-Aldrich Chemie Gmbh, Munich, Germany) for 30 min under ultraviolet light. After washing in ddH_2_O, unreacted silver was discarded by incubation with 5% sodium thiosulfate for 5 min. Wells were washed again, and images were taken using bright field microscopy.

### 4.10. RNA Isolation, cDNA Synthesis and Quantitative PCR

After cultivation for 7 days following the respective protocols for chondrogenesis and osteogenesis as well as after 3 weeks in terms of osteoclastogenesis, cells/cell pellets were harvested, washed with PBS and RNA isolation was performed. Total RNA was extracted using the Arcturus™ PicoPure™ RNA Isolation Kit (Thermo Fisher Scientific, Waltham, MA, USA) according to the manufacturers’ instructions. RNA was stored at −80 °C until further processing. The cDNA was synthesized by reverse transcription using Sensiscript^®^ Reverse Transcription Kit (QIAGEN GmbH, Hilden, Germany). cDNA was stored at −20 °C until further processing. RNA-expression was analyzed using the DyNAmo Flash SYBR Green qPCR Kit at a Stratagene Mx3000P and the Stratagene Mx3000P (Agilent Technologies, Santa Clara, CA, USA) following our standard protocol: 7 min at 95 °C (initial denaturation); 45 cycles of 5 s at 95 °C (denaturation), 7 s at 60 °C (primer annealing), 9 s at 72 °C (elongation); stepwise increase of the temperature from 50 to 98 °C every 30 s (melting curve). Primers were purchased from TIB Molbiol (Berlin, Germany) and are listed in Table 1. Data were normalized to the gene expression of elongation-factor-1α1 (*EEF1A*), using the ΔCt-method.

### 4.11. Statistical Analysis

Statistical analyses were carried out using GraphPad^®^ Prism software (GraphPad Software, La Jolla/San Diego, CA, USA). All values are expressed as the mean ± SEM if not indicated otherwise. One and two-way ANOVA were performed where appropriate including Bonferroni’s multiple comparison post-hoc test. Mann–Whitney U-test was applied for independent datasets while dependent datasets were compared by means using the Wilcoxon-signed rank test. Values of *p* < 0.05 were considered statistically significant.

## Figures and Tables

**Table 1:** Primer list.

Gene Symbol	Gene	Sequence of Forward Primer	Sequence of Reverse Primer
*ACAN*	Aggrecan	AACGCAGACTACAGAAGCGG	GGCGGACAAATTAGATGCGG
*ACP5*	Acid phosphatase 5, tartrate resistant	CTTTGTAGCCGTGGGTGACT	GGGAGCGGTCAGAGAATACG
*COL1A1*	Collagen type I alpha 1 chain	CAGCCGCTTCACCTACAGC	TTTTGTATTCAATCACTGTCTTGCC
*COL2A1*	Collagen type II alpha 1 chain	GAGCCAAAGGATCTGCTGGT	TTGGGGCCTTGTTCACCTTT
*EEF1A1*	Elongation factor 1-alpha 1	GTTGATATGGTTCCTGGCAAGC	TTGCCAGCTCCAGCAGCCT
*RANK*	Receptor activator of NF-κB	ATGGTGGGCTACCCAGGTGA	ACTTGCGGCTGCACAGTGA
*RUNX2*	Runt-related transcription factor 2	TTACTTACACCCCGCCAGTC	TATGGAGTGCTGCTGGTCTG
*SOX9*	SRY-box transcription factor 9	CGCCTTGAAGATGGCGTTG	GCTCTGGAGACTTCTGAACGA
